# Manifestation of Donor–Acceptor Properties of N-Doped Polymer Carbon Dots During Hydrogen Bonds Formation in Different Solvents

**DOI:** 10.3390/polym16243585

**Published:** 2024-12-21

**Authors:** Anisiya Korepanova, Kirill Laptinskiy, Tatiana Dolenko

**Affiliations:** 1 Department of Physics, Moscow State University, 119991 Moscow, Russia; laptinskiy@physics.msu.ru (K.L.); tdolenko@mail.ru (T.D.); 2 Skobeltsyn Institute of Nuclear Physics, Moscow State University, 119991 Moscow, Russia

**Keywords:** polymer carbon dots, absorption spectroscopy, photoluminescence, acidity, basicity, photoluminescence quantum yield, photoluminescence lifetime, hydrogen bonds, donor–acceptor properties

## Abstract

The effective use of polymer carbon dots (PCD) in various fields of science and technology requires a more detailed understanding of the mechanisms of their photoluminescence formation and change as a result of their interaction with the environment. In this study, PCD synthesized via a hydrothermal method from citric acid and ethylenediamine are studied in various solvents using FTIR spectroscopy, optical absorption spectroscopy, and photoluminescence spectroscopy. As a result of the analysis of the obtained dependencies of such PCD spectral characteristics as the photoluminescence FWHM, the photoluminescence quantum yield, the photoluminescence lifetime on the acidity and basicity of the solvent, a hypothesis was formulated on the formation mechanism of hydrogen bonds between the PCD surface groups and the molecules of the environment, and conclusions were made about the donor–acceptor nature of the synthesized PCD.

## 1. Introduction

In recent years, creating a method for rapid and effective diagnostics of various biological and technological environments at the molecular level has become one of the major tasks for many areas of science and industry. In the field of nanotechnology, research is aimed at developing sensors based on various nanomaterials for determining the physicochemical parameters of the environment. Carbon dots (CD) are a class of carbon nanomaterials that are extremely promising for the creation of optical nanosensors due to a unique set of properties: the presence of stable intense photoluminescence (PL) (the luminescence quantum yield is, on average, tens of percent) in the visible range, sensitive to changes in the state of the environment, the ability to achieve deliberate surface modification (leading to a change in the optical properties of the CD), biocompatibility, and simplicity of synthesis [1,2,3,4,5]. Spectral characteristics of CD PL (such as PL intensity, PL band width, and maximum position) are highly sensitive to environment changes and depend on the properties of the environment with the molecules of which they interact [6,7,8,9,10,11,12]. In the study [6], based on the results of PL quenching of carbon quantum dots, the concentrations of broad-spectrum antibiotics, phenol derivatives, and some types of organic solvents in water were determined. The authors of [7,8] developed a photoluminescent nanosensor based on CD for the simultaneous concentration determination of three metal cations and one anion in multicomponent liquid media using neural networks.

The combination of intense and environment-sensitive PL of nanoparticles, biocompatibility, non-toxicity, and hydrophilic/hydrophobic surface allows the use of CD not only for the analysis of technological environments [7,8], but also for solving various biomedical problems, such as pH measurement [13,14], visualization [10,11,15,16], temperature monitoring [10], and composition diagnostics [11] of biological tissues. The authors of the publication [11] used CD to visualize *E. coli* and *Staphylococcus aureus* in biological tissue. In addition, the synthesized CD have proven to be an effective enhancing agent for photodynamic therapy, which aims to destroy bacterial cell membranes. The authors of [12] discovered that CD activate the adsorption properties of hydrogels, which contributes to more efficient removal of harmful substances from biological fluids.

Despite the fact that the possible implementations of CD are already being actively studied in various fields of science and technology, a consensus on the nature and source of their photoluminescence has not been reached. Studying the mechanisms of CD photoluminescence formation is a no less important task than developing their practical applications. According to published data, CD photoluminescence can originate both from the carbon core and from numerous functional groups in the composition of the CD surface [17,18,19,20,21,22]. For example, in the paper [22], relying on the analysis of PL spectra and Raman scattering of light by suspensions of carbon nanoparticles, it was shown that the influence of the solvent on the PL of nanodiamonds and CD is largely determined by the strength and number of hydrogen bonds that solvent molecules can form with the surface groups of nanoparticles. Taking into account the locality of hydrogen bonds, it can be concluded that the source of photoluminescence of the carbon nanoparticles considered in the study are surface states. According to the authors’ explanation, the observed effects are due to different degrees of change in the population of the upper excited levels of the surface groups of nanoparticles under the influence of hydrogen bonds. At the same time, it should not be forgotten that, in addition to the influence of the solvent on the optical properties of carbon nanoparticles, the nanoparticles themselves influence the properties of the solvent, and the nature of the influence is determined by the surface of the nanoparticles [23,24]. It is obvious that effective use of CD cannot be achieved without detailed study of the mechanisms of formation and dependence of their photoluminescence on interactions with the environment. An important stage of this study is exploring the effect of various environmental parameters, including solvent parameters, on the spectral characteristics of CD PL.

The results from many authors available to date indicate that the solvent is one of the determining factors influencing the optical properties of CD (photoluminescence quantum yield (PLQY), shape, intensity, and position of the absorption and PL bands, PL lifetime, etc.) [25,26,27,28]. The authors of [25] conducted a comparative analysis of the effect of solvents on the position of absorption bands corresponding to electronic transitions in different excited states of CD. The results of measuring the PL lifetimes showed that surface states have the shortest PL lifetimes and are most susceptible to quenching with solvent molecules, as compared to the carbon core states. It is important to note that the composition and structure of CD also affect the nature of the change in their optical properties depending on the solvent. In [26], carbon nanoparticles were separated into CD and a molecular fluorophore using dialysis and column chromatography. It was discovered that the CD PLQY decreased, and the PLQY of the molecular fluorophore increased with increasing solvent polarity. The same solvents can lead to PL quenching of some fluorophores and to enhancement of the PL of others, depending on the chemical structure of these fluorophores. The authors of [27] proposed the following mechanism for the influence of a combination of intramolecular charge transfer processes and the formation of hydrogen bonds during the interaction of carbon quantum dots (CQD) with solvents on the optical properties of CQD. In protic solvents, surface groups of CQD form a strong network of hydrogen bonds with solvent molecules, which can lead to the stabilization of the excited state and a decrease in the contribution of intramolecular charge transfer and, thus, increase the red shift in the CQD PL. Furthermore, the hydrogen bond network can facilitate the fixation of the CQD surface groups and, thus, reduce the energy losses due to rotational and vibrational transitions, which results in an increase in CQD PLQY in protic solvents. The authors of [28] analyzed the dependence of the CD PL Stokes shift on the orientational polarizability of the solvents in the Lippert–Mataga approximation and established that CD form strong hydrogen bonds with molecules of solvents belonging to the class of alcohols.

Despite the large number of publications focusing on the study of the solvent influence on the optical properties of CD, there are very few studies that explore the relationship between the photoluminescent characteristics of CD, and important parameters such as acidity SA and basicity SB of solvents introduced by Catalan [29]. The acidity of a molecular compound determines its ability to give up charge, including being a donor for the formation of hydrogen bonds. In turn, basic substances possessing the ability to attach hydrogen atoms serve as acceptors of hydrogen bonds [30]. The SA value characterizes the donor properties of the solvent molecules, while the SB value—the acceptor properties of its molecules. Photoluminescent dyes as a class of luminescent materials have been studied for much longer than CD, therefore the acidity and basicity parameters SA and SB can be found in many studies on the interactions of photoluminescent dyes with the environment [31,32,33,34,35]. Thus, in articles [31,32,33,35], the method of multiple linear regression was used to calculate which of the solvent parameters (SA and SB were considered among others) make the greatest contribution to the change in the spectral characteristics of the dye. Based on such calculations, it is possible to determine the nature of the interaction of individual dye luminophores with solvent molecules (general or specific interaction).

The results of many studies show that the PL formation mechanisms of photoluminescent dyes are similar to the PL formation mechanisms of molecular fluorophores contained in CD [36,37]. Therefore, for our deeper understanding of the nature of CD PL, it is very important to study the effect of the acidity and basicity solvent parameters on the optical properties of CD. Moreover, when analyzing the results obtained for polymeric CD solutions in this paper, we will use approaches that have ensured the effectiveness of dye solutions studies.

In this paper, the polymer carbon dots (PCD) photoluminescence formation mechanisms are studied based on the dependencies of the photoluminescence spectral characteristics of PCD, synthesized via a hydrothermal method from citric acid and ethylenediamine at different ratios of precursors on the acidity and basicity of solvents. An extensive comprehensive analysis of the simultaneous effect of solvent acidity and basicity on the change in the optical properties of PCD has been carried out by a set of different spectroscopic methods and described in more detail than in the available papers. The relationship between the precursor ratio of CD and the nature of their interaction with the solvent was also considered for the first time.

## 2. Materials and Methods

### 2.1. Synthesis of PCD and Preparation of Samples

The study objects in this paper are PCD synthesized hydrothermally from citric acid (CA) and ethylenediamine (EDA) at a temperature of 180 °C for 3 h with the following precursor ratios: EDA:CA = 0.1; 0.5; 1; 2; 4; 10; 20. EDA is a source of nitrogen for PCD doping, therefore, with an increase in the EDA concentration in the precursor mixture, the number of nitrogen-containing functional groups in the PCD composition increases. The synthesis and characterization of PCD are described in detail in paper [17]. It should be noted that our study considers a specific class of PCD that are formed under the specified conditions of hydrothermal synthesis [38]. The synthesized polymer carbon dots are the result of polymerization and then the beginning of carbonization of the mixture of precursor molecules. The obtained objects of study are polymer fragments rather than particles with a distinct carbon core.

The measurements of size by dynamic light scattering showed that all PCD synthesized under the listed conditions are characterized by a size of 1.1 ± 0.3 nm. Taking into account that for different carbon allotropes the average interatomic distance is ~1.47 Å, and the size of a carbon atom is ~0.9 Å, it can be estimated that, in the roughest approximation, the synthesized study objects consist of approximately 10 carbon atoms. Thus, it can be concluded that the synthesized PCD are relatively small organic molecular polymer complexes. Depending on the ratio of precursors, the synthesized PCD were characterized by different values of the zeta-potential. At the ratio of precursors EDA:CA = 0.1:1, the value of the zeta-potential was +1.7 ± 3.9 mV, and for the nitrogen-rich PCD sample (EDA:CA = 20:1), the value of the zeta-potential was negative and was −17.0 ± 5.5 mV.

The following solvents were used to prepare the PCD solutions: deionized water (Millipore Simplicity UV water purification system, Merck, MA, USA), methanol, ethanol, benzyl alcohol, isopropanol, dimethyl sulfoxide (DMSO), ethyl acetate, and acetone. The concentration of PCD in all solutions was 94 mg/L. All chemical reagents used in the study were of analytical grade or higher. The solvents for this study were selected so that the parameters characterizing their acidic and basic properties differed significantly from each other (Table 1). The values of these parameters were acquired from the empirical Catalan scale [29].

### 2.2. Experimental Methods

The prepared solutions with the same concentration (equal to 94 mg/L) of each type of PCD in all the listed solvents were studied by FTIR absorption spectroscopy, optical absorption spectroscopy, and photoluminescence spectroscopy.

FTIR absorption spectra were recorded using a Bruker Invenio R Fourier spectrometer equipped with an ATR accessory with a diamond crystal in the 500–4000 cm^−1^ range with a resolution of 1 cm^−1^. Sample preparation and spectra acquisition consisted of the following: a drop (the drop volume was 2 μL) of an aqueous solution of PCD was placed on a diamond crystal of the ATR accessory of the FTIR spectrometer. Then, for 10 min (preliminary experiments showed that such time of air exposure achieved complete drying of the sample at an average room humidity of about 39%—the FTIR spectra did not change with further drying) the drop was dried in a constant air flow, and the FTIR spectrum of the resulting powder was recorded. The powder obtained on the crystal was “washed” with the appropriate solvent: five drops (the volume of each drop was 1 μL) of the solvent were successively applied, between the application of each drop, the sample was dried for 4 min.

Optical density spectra of PCD solutions were obtained using a two-channel Shimadzu UV-1800 spectrophotometer in the 200–500 nm range with a step of 1 nm at an average scanning rate. A quartz cell with a solvent was placed in the reference channel; the optical path length was 10 mm.

PL spectra of PCD solutions were recorded on a Shimadzu RF-6000 spectrofluorimeter under excitation in the 200–450 nm wavelength range. PL emission spectra were measured in the wavelength range from 350 to 650 nm with a step of 1 nm at a scanning rate of 12,000 nm/min and low sensitivity. The samples were placed in quartz cells with an optical path length of 10 mm.

Photoluminescence quantum yield of PCD solutions was determined using the reference dye method [39]. Quinine sulfate in a 0.05 M/L aqueous solution of sulfuric acid was used as a reference dye (the value of PLQY upon excitation at a wavelength of 350 nm is 58% [39]).

PCD photoluminescence decay kinetics were obtained using a setup consisting of a picosecond laser (excitation wavelength 375 nm, pulse repetition frequency 5 MHz, pulse duration 40 ps), a DSC900PS single photon counting system (Zolix), and an OmniFluo 900 LPS spectrofluorimeter (Zolix). The kinetic curves were recorded at a PL emission wavelength of 440 nm.

## 3. Results and Discussions

### 3.1. FTIR Absorption Spectroscopy of Synthesized Polymer Carbon Dots

**Figure 1:**
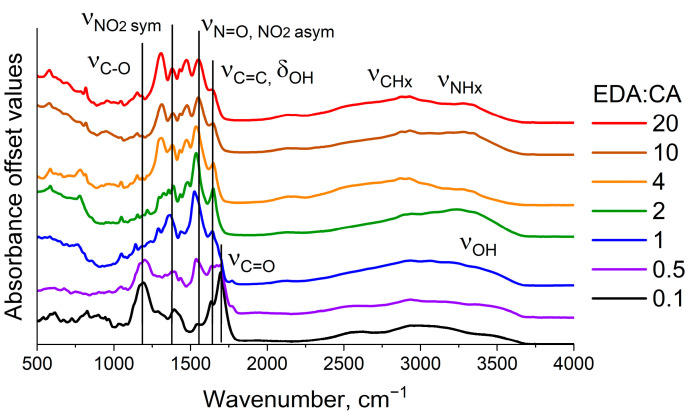
FTIR absorption spectra of PCD powders with varying nitrogen content evaporated from water.

To determine the structural features of the synthesized PCD, FTIR absorption spectra of powders of seven types of PCD evaporated from water were recorded. These spectra are presented in Figure 1.

As one can see from Figure 1, PCD FTIR absorption spectra have a distinct set of absorption bands corresponding to vibrational transitions of various functional groups on their surface [40]. However, it can be observed that the composition of the absorption bands varies depending on the EDA and CA ratio. One CA molecule contains 3 carboxyl groups in its composition, while an EDA molecule contains 2 amino groups. The molar equality of precursors in the synthesis reaction is achieved when there is one amino group per carboxyl group, that is, when EDA:CA = 3:2 = 1.5. On the basis of this ratio of precursors, synthesized PCD can be divided into 2 classes: nitrogen-deficient PCD1 (EDA:CA = 0.1, 0.5, 1) and nitrogen-rich PCD2 (EDA:CA = 2, 4, 10, 20). The bands near 3330 cm^−1^ and 2870 cm^−1^ are attributed to the stretching vibrations of amino groups -NH and methylene groups -CH, respectively [41]. A broad stretching vibrations band of hydroxyl groups -OH is located in the 3000–3600 cm^−1^ region. The stretching vibrations of C=O (1710 cm^−1^) and C-O (1200 cm^−1^) of carboxyl groups -COOH are present in the spectra of PCD1 and absent in the spectra of PCD2 [42]. The bands at 1540 cm^−1^ and 1375 cm^−1^ are assigned to the asymmetric and symmetric stretching vibrations of nitro groups -NO_2_, respectively [43]. When transitioning from the class of PCD1 solutions to the class of PCD2 solutions, the intensity of nitro group bands decreases, nitro groups lose oxygen and are converted into nitroso groups -N=O (1540 cm^−1^). The band at 1650 cm^−1^, caused by bending vibrations of -OH groups of residual water in PCD1, is transformed into a stretching vibrations band of non-aromatic C=C bonds upon transitioning to PCD2. The functional groups found in the PCD surface composition are characterized by a high degree of polarity, which indicates the ability of PCD fluorophores to form strong hydrogen bonds with molecules of the environment.

### 3.2. Optical Absorption and Photoluminescence Spectroscopy of Polymer Carbon Dots in Different Solvents

**Figure 2:**
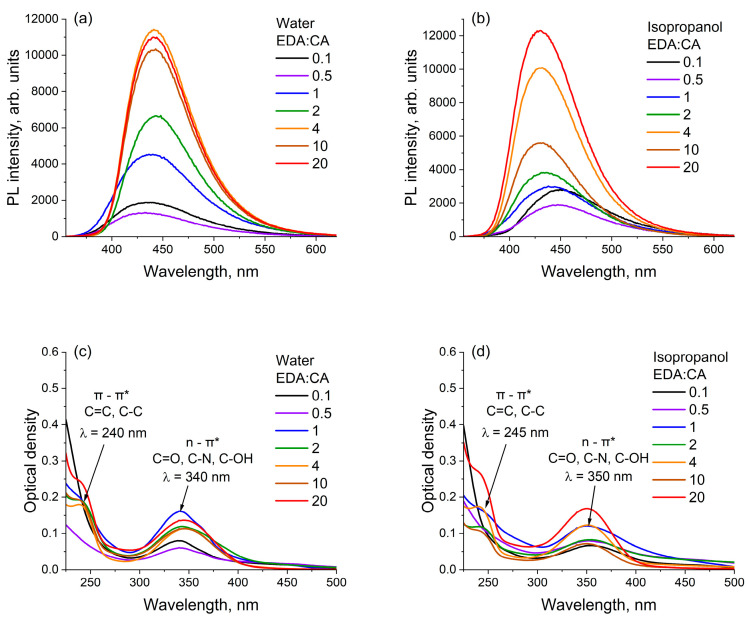

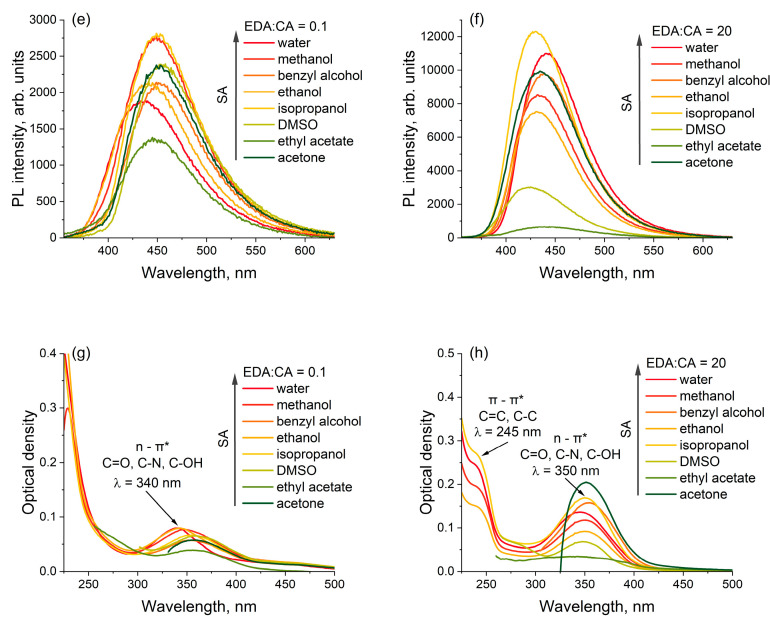
PL and optical absorption spectra of all PCD in water (**a**,**c**) and isopropanol (**b**,**d**), and PL and optical absorption spectra of PCD with EDA:CA = 0.1 (**e**,**g**) and EDA:CA = 20 (**f**,**h**) in all the studied solvents.

Figure 2 demonstrates photoluminescence and optical absorption spectra of all seven types of PCD in the most acidic (water) (Figure 2a,c) and most basic (isopropanol) (Figure 2b,d) solvents, as well as photoluminescence and optical absorption spectra of PCD with the lowest (Figure 2e,g) and highest (Figure 2f,h) nitrogen content in all solvents. It is evident that the spectra have several features. The intensity of PL, the width and shape of PL bands, optical absorption, and PL peak positions depend significantly on both the solvent and the nitrogen concentration in the PCD.

For further discussion of the influence of solvent parameters—primarily polarity—on the optical properties of PCD, it is necessary to understand whether the interaction of the synthesized PCD with solvent molecules is general or specific. To assess the influence of solvent polarity on the emission of fluorophores in solution, the orientational polarizability parameter of the solvent ∆*f* is used, which explains the spectral shifts caused by the solvent dipoles reorientation and the electron density redistribution in its molecules [39]. This parameter is calculated using the Lippert equation based on the permittivity ε and the refractive index *n* of the medium [39]:

(1) $$∆f=\frac{\varepsilon -1}{2\varepsilon +1}-\frac{n^{2}-1}{{2n}^{2}+1}.$$

Based on the degree of linearity of the dependence of fluorophores, PL Stokes shift in different solvents ∆ν ($\nu_{A}$—absorption peak position, $\nu_{PL}$—PL peak position) on the solvent polarity function ∆f, which is described by the Lippert–Mataga Equation (2), one can conclude whether there are specific interactions between fluorophores and solvent molecules [44,45,46]:

(2) $$∆\nu =\nu_{A}-\nu_{PL}=\frac{2}{hc}\left(\frac{\varepsilon -1}{2\varepsilon +1}-\frac{n^{2}-1}{2n^{2}+1}\right)\frac{{\left(\mu_{e}-\mu_{g}\right)}^{2}}{a^{3}}+const.$$

In Equation (2) h is Planck’s constant, c is the speed of light in vacuum, $\mu_{e},\;\mu_{g}$ are the fluorophore dipole moment in the excited and ground states, respectively, and a is the radius of the cavity in which the fluorophore is located.

**Figure 3:**
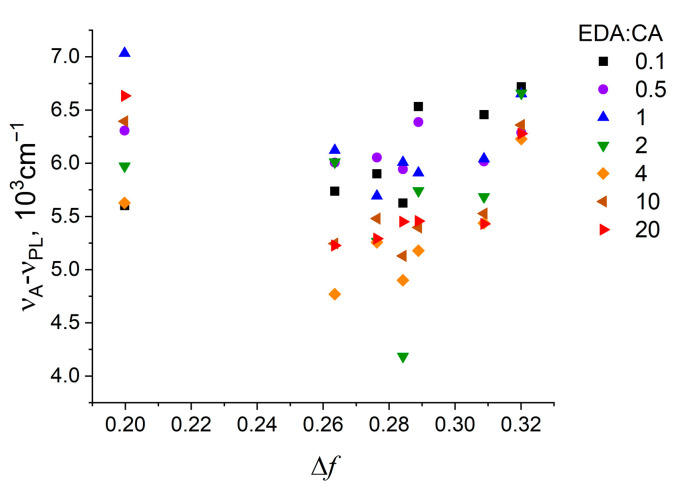
Dependence of the PL Stokes shift on the orientational polarizability of the solvent for all samples. The values of Δ*f* are presented in Table 1.

Figure 3 shows the dependence of the PL Stokes shift on the orientational polarizability of the solvent for all samples. The dependence under consideration is far from linear, which means that specific interactions (e.g., the formation of hydrogen bonds) predominate in the PCD-solvent system [39].

**Figure 4:**
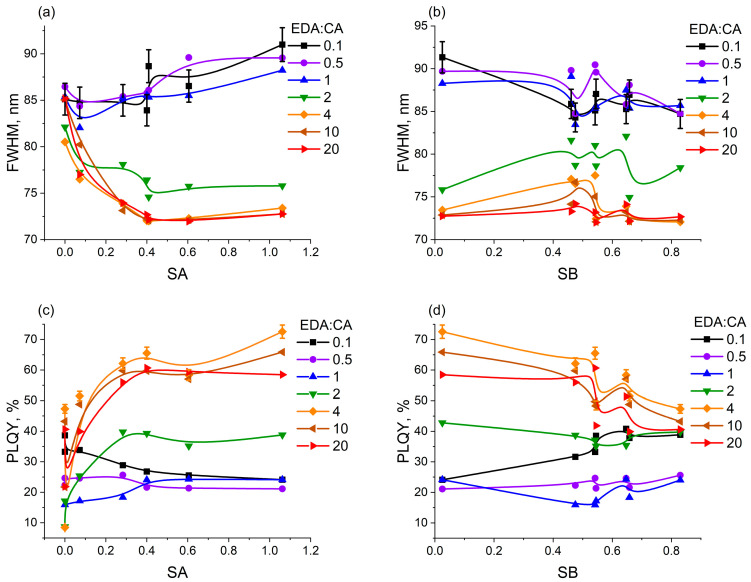
Dependencies of the PCD photoluminescence FWHM and PLQY on the acidity (**a**,**c**) and basicity (**b**,**d**) of the solvent. The errors in the calculated PLQY and measured PL FWHM are 3% and 2% of the corresponding values of these parameters for all PCD solutions.

To study the mechanisms behind the changes in the spectral characteristics of polymer carbon dots under the influence of both the solvent and the concentration of precursors, the dependencies of the PCD photoluminescence FWHM on the acidity SA and basicity SB of the solvents for all seven samples were constructed (Figure 4a,b). The dependencies of the PCD photoluminescence quantum yield on SA and SB were also calculated and constructed (Figure 4c,d).

It is evident from Figure 4a that the photoluminescence FWHM values of all PCD samples are relatively close and are in a narrow range during the interaction of PCD fluorophores with solvents with the lowest acidity (acetone, ethyl acetate, and DMSO). With an increase in the solvent acidity, the PL FWHM of PCD1 class solutions broadens and the PL FWHM of PCD2 class solutions narrows. The opposite result is observed for the dependence of the FWHM of the PL band on the solutions basicity SB (Figure 4b): the PCD1 spectra narrow and the PCD2 spectra broaden with an increase in the solvent basicity. Figure 4c,d show that the PCD1 PLQY decreases and the PCD2 PLQY increases with the rise in solvent acidity, while with the rise in basicity, the PCD1 PLQY increases and the PCD2 PLQY decreases. It can be noticed that the obtained values of PLQY are consistent with the previously published results [47,48]: in the aqueous solutions, PCD made from citric acid and ethylenediamine are characterized by values ~60%.

It is known from studies of photoluminescent dyes [49,50] that the band width of photoluminescence can indicate the relaxation nature of the excited state of fluorophores. Fluorophores that are able to form stronger hydrogen bonds with solvents have wider PL bands. When CD form hydrogen bonds with the molecules of the environment, the surface structure of CD is fixed, therefore, the energy losses for rotational and vibrational intramolecular transitions are significantly reduced [51,52,53], and new PL channels are opened. At the same time, the formation of hydrogen bonds between CD and solvent molecules induces the development of new channels of nonradiative relaxation of the excited state as a result of intermolecular charge transfer. Due to this, the total number of luminescent fluorophores in each emission channel decreases, which leads to a decrease in the PLQY. As a result, the PL bands are wider and less intense than in the case of weak hydrogen bonds. The similarity between the obtained dependencies of the PL FWHM on the PLQY of solutions of both PCD classes may reflect the similarity of mechanisms of change in the photoluminescence of PCD1 and PCD2 in different solvents.

According to the available studies [36,37], there is a similarity between the chemical and optical properties of CD fluorophores and photoluminescent dyes, so the mechanism of changing optical properties depending on the donor–acceptor characteristics of the solvent can be similarly interpreted in the case of both PCD classes. Hydrogen bonds between CD and solvent molecules can be formed in two cases: during the interaction of a basic solvent with fluorophores acting as donors of hydrogen bonds (acidity centers), and during the interaction of an acidic solvent with fluorophores acting as acceptors of hydrogen bonds (basicity centers) [30]. Since the PL spectra of PCD1 class solutions broaden with increasing solvent acidity, while the PLQY of PCD1 decreases, PCD1 form more hydrogen bonds with more acidic solvents. It follows from this that PCD1 fluorophores interacting with solvents are basicity centers. Similarly, PCD2 fluorophores are acidity centers. Thus, nitrogen-rich molecular fluorophores are more likely to be hydrogen bond donors, while nitrogen-deficient fluorophores are more likely to be hydrogen bond acceptors.

### 3.3. Study of PCD Photoluminescence Lifetimes in Different Solvents

**Figure 5:**
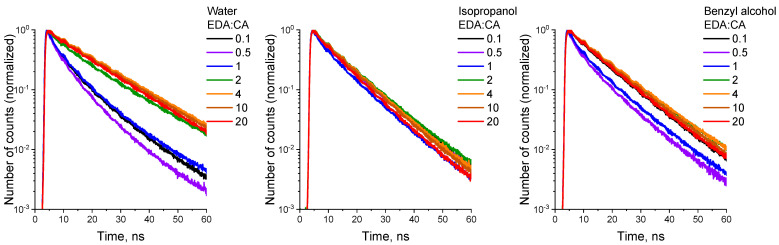
PCD photoluminescence decay kinetics in different solvents.

The next step in the study of the interaction between PCD and solvents was obtaining photoluminescence decay kinetics of both classes of synthesized PCD in all solutions. For the approximation of the decay curves (Figure 5) and the calculation of the PCD PL lifetimes, a biexponential model was used [54].

As a result of the PL decay kinetics analysis of the synthesized PCD1 and PCD2 in aqueous solutions, two fluorophores were allocated: long-lived fluorophore F1 (τ_1_ = 10.6 ± 0.3 ns and 16.4 ± 0.9 ns for PCD1 and PCD2, respectively), and short-lived fluorophore F2 (τ_2_ = 2.3 ± 0.1 ns and 8.2 ± 0.5 ns for PCD1 and PCD2, respectively). Both fluorophores allocated from PCD2 are longer-lived than that from PCD1.

**Figure 6:**
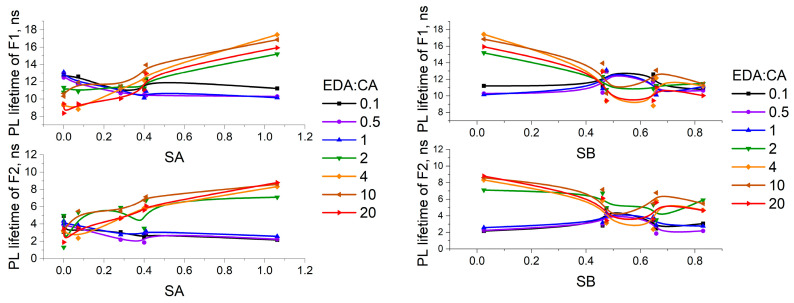
Dependencies of the PCD PL lifetimes on the acidity and basicity of the solvents (errors in the calculated PCD PL lifetimes are 0.3% of the calculated values for all solutions).

Figure 6 shows the dependencies of the PCD fluorophores F1 and F2 photoluminescence lifetimes on the acidity and basicity parameters of the solvents. It follows from the presented data that the PCD2 fluorophores PL lifetimes are more sensitive to the change in solvent than the PCD1 fluorophores PL lifetimes. Significant quenching of PCD2 fluorophores PL is observed upon excitation in a more basic and less acidic medium. On the contrary, in the case of PCD1, the PL lifetimes in a more basic medium exceed the PL lifetimes in a more acidic medium. Hydrogen bonds that PCD form with solvent molecules play an important role in the quenching of PCD photoluminescence. As was mentioned earlier, the formation of hydrogen bonds with solvent molecules fixes the PCD surface structure. As a result, the probability of some nonradiative processes (for example, intermolecular charge transfer) that enable a transition from the excited state to the ground state without emitting a photon of light increases, and the number of luminescent fluorophores decreases. Thus, the overall observed lifetime of specific fluorophore types lessens. The decrease in the PCD1 fluorophores PL lifetimes with a rise in solvent acidity and the decrease in the PCD2 fluorophores PL lifetimes with a rise in solvent basicity support the hypothesis of the acceptor nature of PCD1 fluorophores and the donor nature of PCD2 fluorophores. The weaker effect of solvent acidity and basicity on the PCD1 fluorophores PL lifetimes indicates that PCD1 fluorophores form fewer hydrogen bonds with the solvent than PCD2 fluorophores.

**Figure 7:**
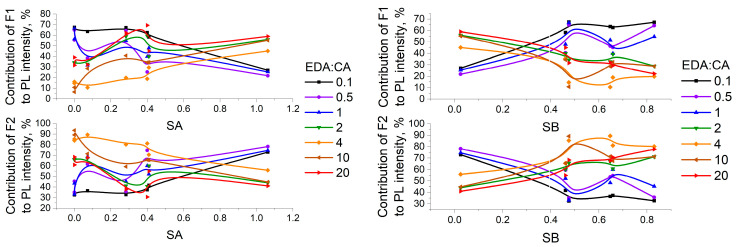
Dependencies of the F1 and F2 percentage contributions into the total PCD PL intensity on the acidity and basicity of the solvent (errors in the calculated contributions are 0.5% of the contributions themselves for all solutions).

The contributions of each fluorophore to the total photoluminescence intensity of all PCD solutions were estimated from the analysis of the biexponential model of PCD PL decay kinetics. The obtained estimates show what percentage of the total number of emitting fluorophores is made up of fluorophores with a certain specific photoluminescence lifetime. Figure 7 demonstrates the dependencies of the F1 and F2 contributions to the total PCD PL intensity on the acidity and basicity of the solvent. It can be seen from Figure 7 that with increasing solvent acidity, the contribution of F1 to the PCD1 photoluminescence intensity decreases, while the contribution of F2 increases. The following occurs for PCD2 solutions: with increasing solvent acidity, the contribution of F1 to the PCD2 photoluminescence intensity increases, while the contribution of F2 decreases. The opposite results are observed when increasing solvent basicity. A decrease in the fluorophore contribution to the total PL intensity indicates a decrease in the number of emitting fluorophores of this type. In the present case, this is due to PL quenching as a result of hydrogen bond formation. The nature of the dependencies shown in Figure 7 suggests that fluorophore F1 forms stronger hydrogen bonds with solvent molecules in PCD1 solutions, and fluorophore F1 forms stronger hydrogen bonds with solvents in PCD2 solutions, i.e., the center of basicity of PCD1 is a longer-lived fluorophore (τ ≈ 10.6 ns), and the center of acidity of PCD2 is a longer-lived fluorophore (τ ≈ 16.4 ns).

### 3.4. Correlations Between the Change in PCD Optical Properties in Different Solvents

To confirm the proposed hypothesis about the donor–acceptor nature of PCD in various solvents, an additional experiment was conducted using FTIR spectroscopy of PCD solutions. As shown above, the FTIR absorption spectra of nitrogen-deficient PCD (class PCD1) contain C=O (1710 cm^−1^) stretching vibrations bands of carboxyl groups –COOH (Figure 1). The position of these bands depends, in particular, on the strength of hydrogen bonds in which C=O participates as an acceptor [55]. The magnitude of the shift determines the strength of hydrogen bonds—the greater the red shift (towards lower wavenumbers), the stronger the hydrogen bond [56].

**Figure 8:**
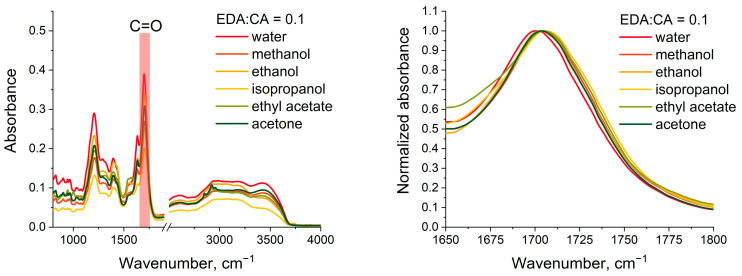
FTIR absorption spectra of PCD (class PCD1) treated with different solvents. On the right is an enlarged fragment of the FTIR spectra in the 1650–1800 cm^−1^ region, normalized to the maximum of the band for visual clarity.

Figure 8 shows the FTIR absorption spectra of PCD1 powders (sample with a precursor ratio of 0.1:1) after the above-described treatment (see Section 2). The study does not present the FTIR absorption spectra of PCD powders dried from DMSO and benzyl alcohol, because these solvents are characterized by a high boiling point (189 °C and 205 °C, respectively) and evaporate extremely slowly under normal conditions. In the FTIR absorption spectra of nitrogen-rich PCD (class PCD2), there are no stretching vibrations bands of carboxyl groups -COOH (Figure 1), therefore PCD2 cannot be studied in this additional experiment.

**Figure 9:**
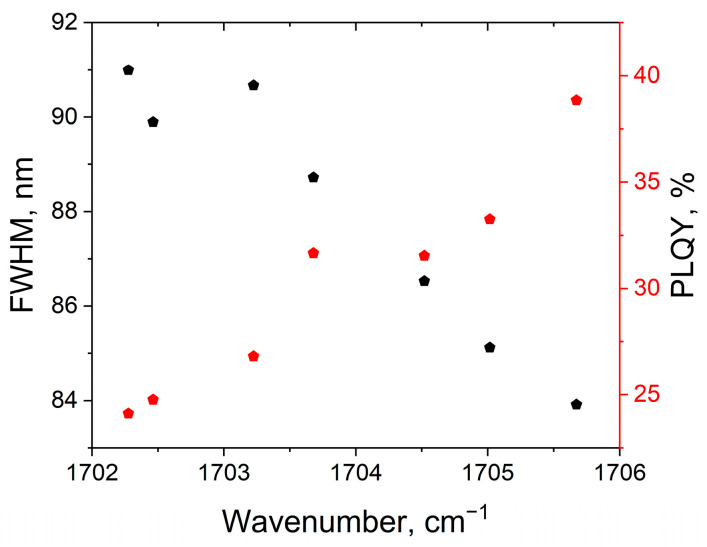
Dependencies of the polymer carbon dots PL FWHM and PLQY on the C=O band peak position in the FTIR absorption spectra for PCD1 solutions (sample with a precursor ratio of 0.1:1).

As can be seen from the presented data, no new bands appear, and no existing bands disappear in the spectra of PCD1 “treated” with different solvents. Firstly, this indicates that new chemical bonds between the PCD functional groups and the solvent molecules are not formed during the interaction of PCD1 with solvents. Secondly, the absence of bands characteristic of solvents indicates that the solvent is not adsorbed in significant quantities by PCD1 surface. Analysis of the C=O band position in the PCD1 spectra shows that when the sample is evaporated from an aqueous solution, the band is located in the lowest wavenumbers region compared to other studied solvents. The greatest shift to a higher wavenumbers region relative to the sample evaporated from water is observed for the sample “treated” with isopropanol. To compare the results of photoluminescence and FTIR spectroscopy, the dependencies of the PCD1 photoluminescence FWHM (sample with a precursor ratio of 0.1:1) and the PLQY of these PCD1 on the C=O band peak position in the FTIR absorption spectra were constructed (Figure 9).

The presented dependencies clearly demonstrate that as the strength of hydrogen bonds decreases (the C=O band peak position shifts to the higher wavenumbers region), an increase in the PCD1 PLQY and a decrease in the PCD1 photoluminescence FWHM are observed.

### 3.5. Hydrogen Bonds Formation Mechanism

Based on the obtained results, the following mechanism of donor–acceptor interaction between the synthesized PCD and the solvent molecules can be formulated. When PCD encounter solvent molecules, the surface functional groups of PCD act either as a donor or as an acceptor of hydrogen bonds with the molecules of the environment. The resulting hydrogen bonds network around PCD fixes the surface electronic structure of PCD, thereby reducing energy losses for rotational and vibrational intramolecular transitions (internal conversion) during the PCD photoluminescence excitation [51,52,53]. Due to this, new, previously non-emitting PL channels open on the PCD surface. At the same time, the formation of hydrogen bonds between PCD and solvent molecules leads to the development of new channels of nonradiative relaxation of the PCD surface groups excited states by means of intermolecular charge transfer through hydrogen bonds [49,50] and scattering of the share of excitation in the solvent. Thus, when hydrogen bonding occurs between PCD and a solvent, the number of emission channels increases, but the number of photons emitted by each of them decreases. In this case, nitrogen-deficient PCD (PCD1) act as an acceptor and enter hydrogen bonds with more acidic solvents, while nitrogen-rich PCD (PCD2) act as a donor and form hydrogen bonds with more basic solvents.

## 4. Conclusions

In the present study, seven types of polymer carbon dots with different precursor ratios were synthesized via a hydrothermal method from citric acid and ethylenediamine (EDA:CA = 0.1; 0.5; 1; 2; 4; 10; 20), and then characterized. Their photoluminescent properties were explored in solvents with different acidity (SA) and basicity (SB) parameters: water, methanol, benzyl alcohol, ethanol, isopropanol, DMSO, ethyl acetate, and acetone.

The analysis of the experimental results obtained by methods of FTIR absorption spectroscopy, optical absorption spectroscopy and photoluminescence spectroscopy, including time-resolved, demonstrated a significant dependence of the spectral characteristics of PCD photoluminescence on the acidity and basicity of the solvent. It was determined that the reason for this dependence is the formation of hydrogen bonds of varying strength between PCD functional groups and solvent molecules. Following the analysis of the obtained dependencies of the PCD photoluminescence FWHM, the PLQY, the PL lifetime and the contribution of individual fluorophores to the total PCD PL intensity on the solvent parameters, conclusions were made about the donor–acceptor nature of the synthesized polymer carbon dots. A hypothesis was formulated about the mechanism of hydrogen bonds influence on the PCD optical properties in various solvents.

It has been established that in solutions with different acidity and basicity, nitrogen-deficient PCD (class PCD1) act as basicity centers, i.e., hydrogen bond acceptors. Moreover, the predominant role in this interaction belongs to the long-lived PCD1 fluorophores F1. Nitrogen-rich PCD (class PCD2) in the studied solvents act as acidity centers, i.e., hydrogen bond donors. The predominant role in this interaction belongs to the long-lived PCD2 fluorophores F1.

The ability to accurately divide synthesized PCD by their donor and acceptor nature is of great importance for the development of PCD practical applications. Recently, the donor–acceptor interaction between nanoparticles and the medium has been actively used to create nanosensors and visualization agents in biomedicine, for example, for the detection of RNA viruses [57] and illicit drugs [58] in the organism. It also proved effective for the selective sensing of cancer cells [59]. The results obtained in this paper open a vast field for future research and development of PCD-based nanosensors and medical agents in various environments. Knowledge of the mechanisms of medium parameters influence on the photoluminescence of carbon nanomaterials allows us to solve many fundamental problems in nanotechnology and material science. For example, the knowledge of the mechanisms of the hydrogen bond formation between the surface of CD and the surrounding molecules (of the solvent, in particular) allows one to optimize pH-sensing mechanism of CD [60]. Another possible application of the presented results is that it may be a valuable “brick” for the formation of the theory of different hydrogen bonding-mediated assembly of CD complexes [61].

## Figures and Tables

**Table 1:** Solvent parameters.

Solvent	SA	SB	Δf
Water—H_2_O	1.062	0.025	0.32
Methanol—CH_4_O	0.605	0.545	0.31
Benzyl alcohol—C_7_H_8_O	0.409	0.461	0.21
Ethanol—C_2_H_6_O	0.400	0.658	0.29
Isopropanol—C_3_H_8_O	0.283	0.830	0.28
DMSO—C_2_H_6_OS	0.072	0.647	0.26
Ethyl acetate—C_4_H_8_O_2_	0	0.542	0.20
Acetone—C_3_H_6_O	0	0.475	0.28

## Data Availability

Data are available on request to the corresponding author.
